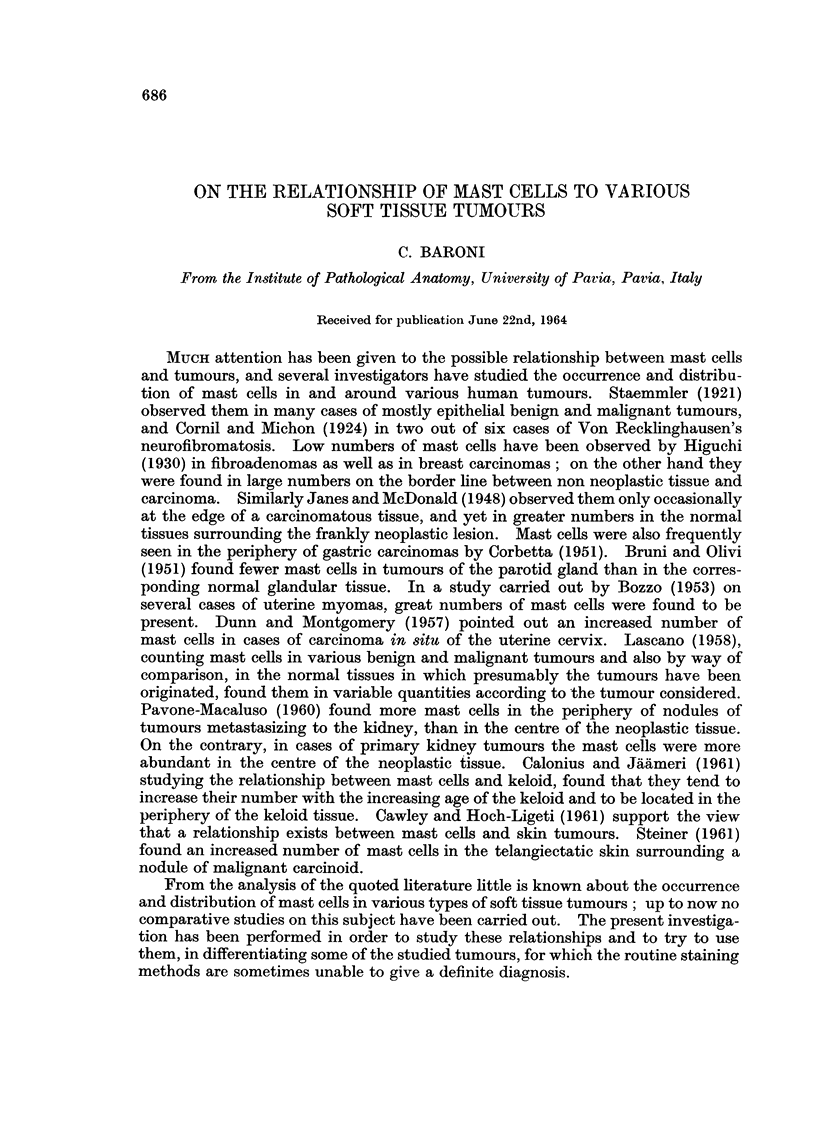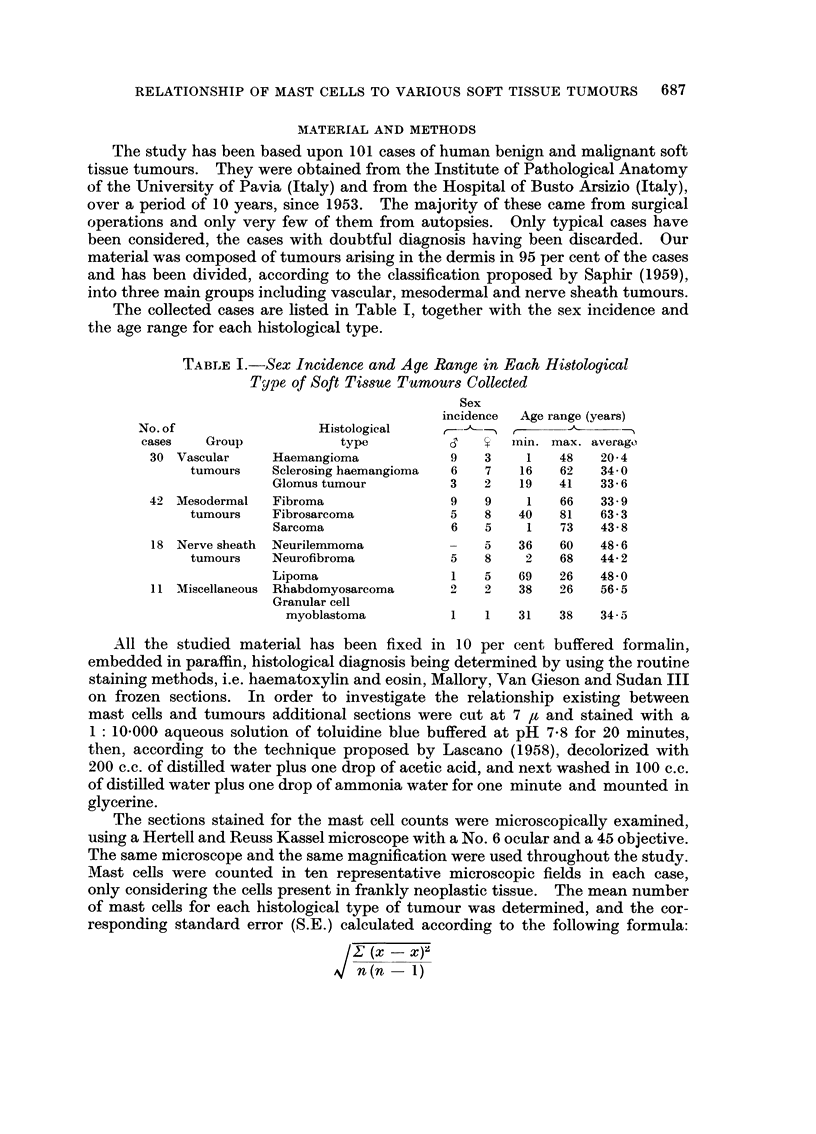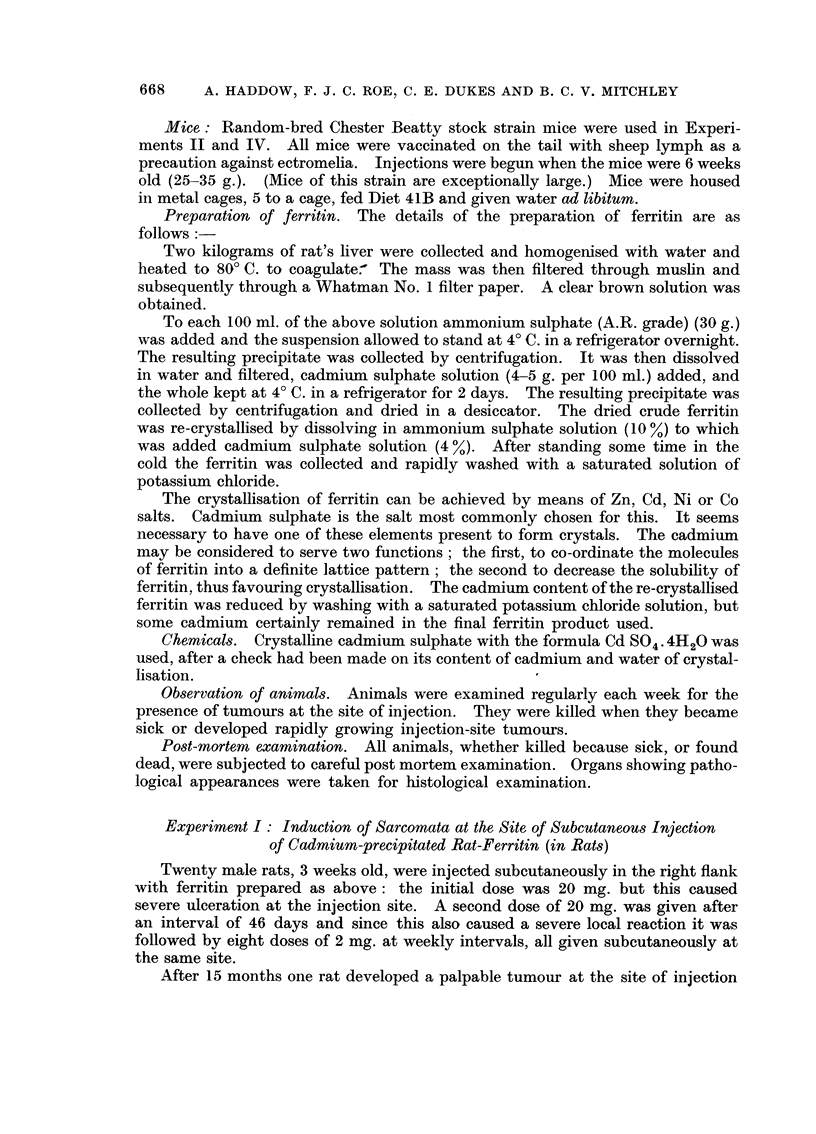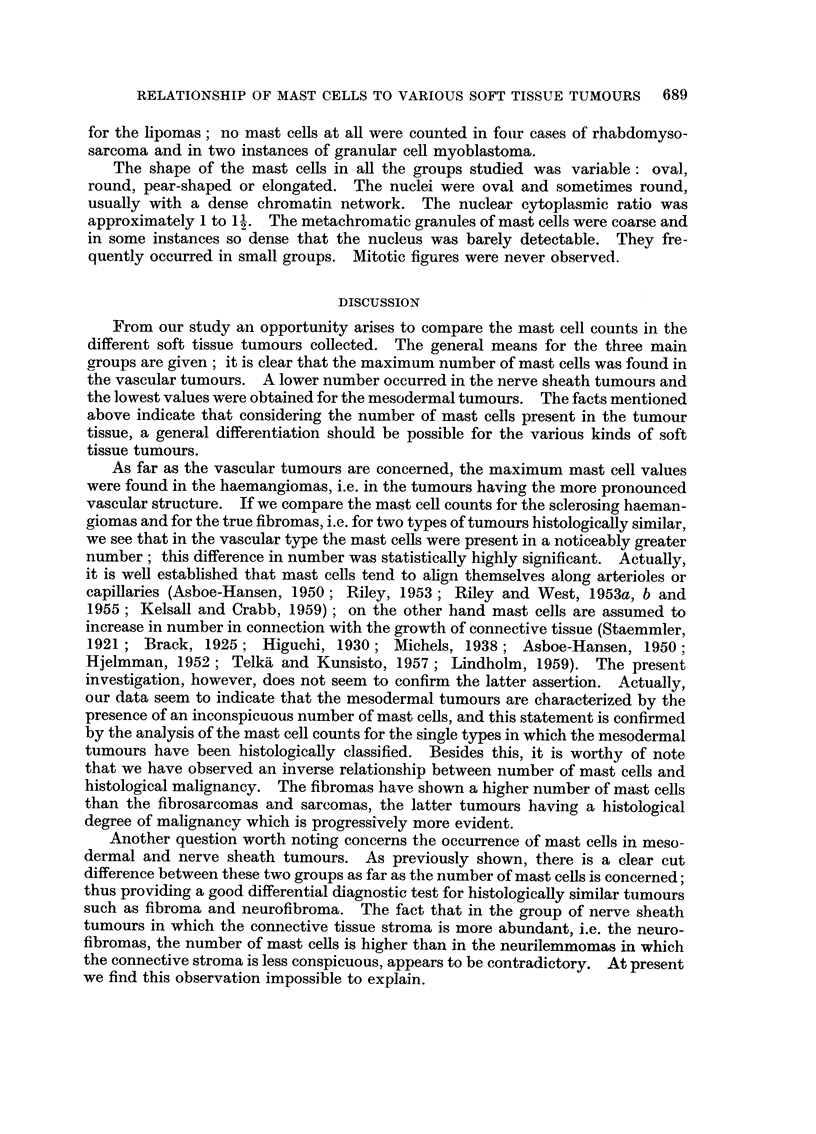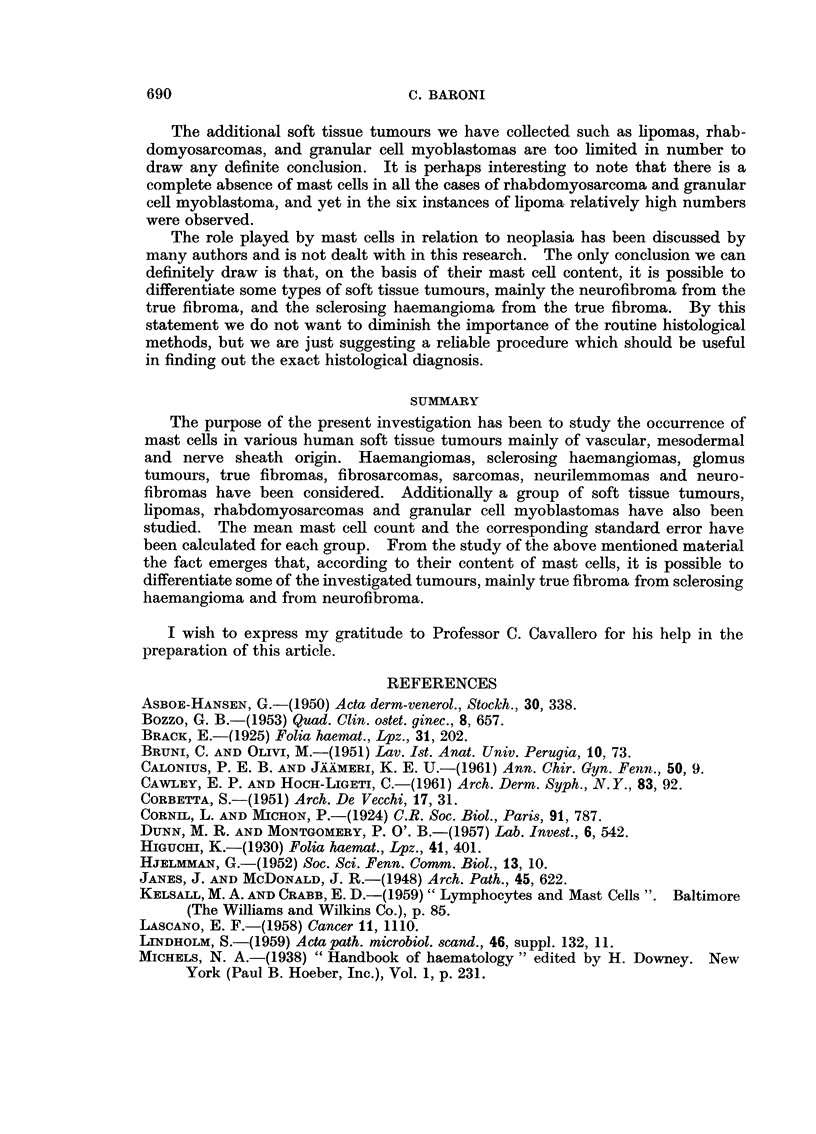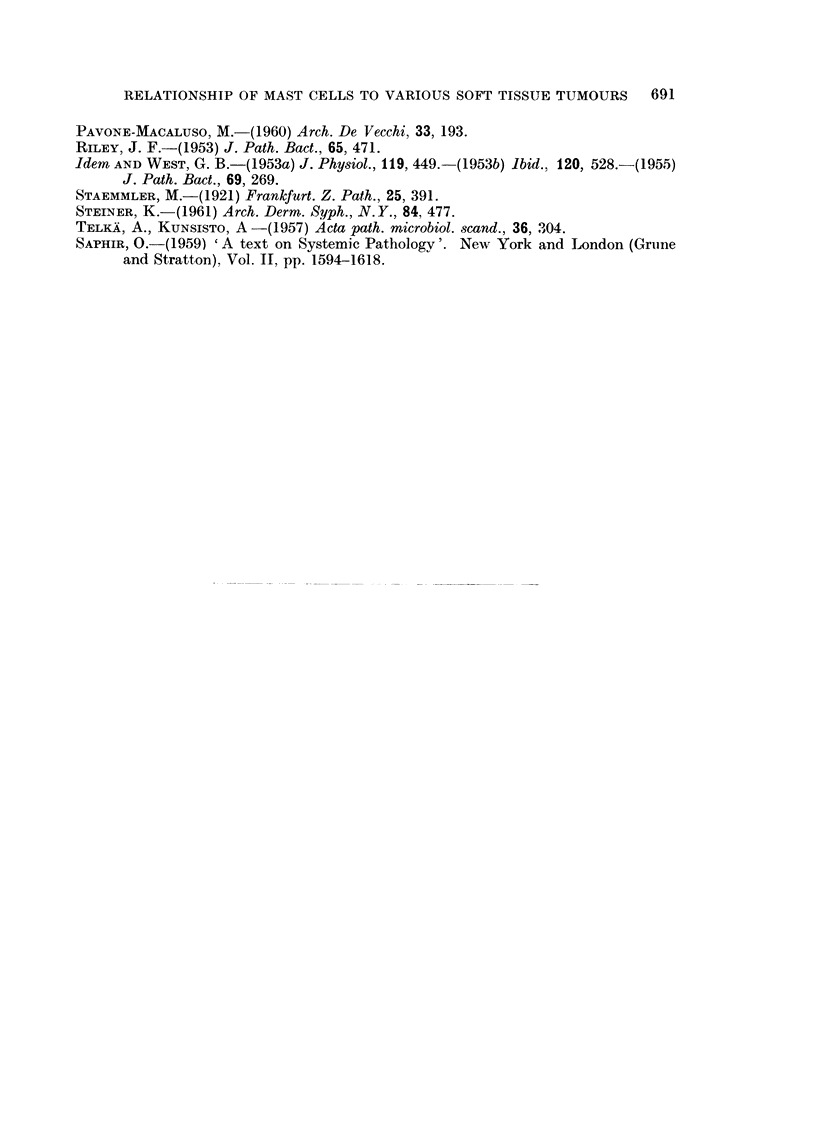# On the Relationship of Mast Cells to Various Soft Tissue Tumours

**DOI:** 10.1038/bjc.1964.79

**Published:** 1964-12

**Authors:** C. Baroni


### ON THE RELATIONSHIP OF MAST CELLS TO VARIOUS SOFT TISSUE TUMOURS

C. BARONI

*From the Institute of Pathological Anatomy, University of Pavia, Pavia, Italy*

Received for publication June 22nd, 1964

MUCH attention has been given to the possible relationship between mast cells and tumours, and several investigators have studied the occurrence and distribution of mast cells in and around various human tumours. Staemmler (1921) observed them in many cases of mostly epithelial benign and malignant tumours, and Cornil and Michon (1924) in two out of six cases of Von Recklinghausen's neurofibromatosis. Low numbers of mast cells have been observed by Higuchi (1930) in fibroadenomas as well as in breast carcinomas ; on the other hand they were found in large numbers on the border line between non neoplastic tissue and carcinoma. Similarly Janes and McDonald (1948) observed them only occasionally at the edge of a carcinomatous tissue, and yet in greater numbers in the normal tissues surrounding the frankly neoplastic lesion. Mast cells were also frequently seen in the periphery of gastric carcinomas by Corbetta (1951). Bruni and Olivi (1951) found fewer mast cells in tumours of the parotid gland than in the corresponding normal glandular tissue. In a study carried out by Bozzo (1953) on several cases of uterine myomas, great numbers of mast cells were found to be present. Dunn and Montgomery (1957) pointed out an increased number of mast cells in cases of carcinoma *in situ* of the uterine cervix. Lascano (1958), counting mast cells in various benign and malignant tumours and also by way of comparison, in the normal tissues in which presumably the tumours have been originated, found them in variable quantities according to the tumour considered. Pavone-Macaluso (1960) found more mast cells in the periphery of nodules of tumours metastasizing to the kidney, than in the centre of the neoplastic tissue. On the contrary, in cases of primary kidney tumours the mast cells were more abundant in the centre of the neoplastic tissue. Calonius and Jäämeri (1961) studying the relationship between mast cells and keloid, found that they tend to increase their number with the increasing age of the keloid and to be located in the periphery of the keloid tissue. Cawley and Hoch-Ligeti (1961) support the view that a relationship exists between mast cells and skin tumours. Steiner (1961) found an increased number of mast cells in the telangiectatic skin surrounding a nodule of malignant carcinoid.

From the analysis of the quoted literature little is known about the occurrence and distribution of mast cells in various types of soft tissue tumours ; up to now no comparative studies on this subject have been carried out. The present investigation has been performed in order to study these relationships and to try to use them, in differentiating some of the studied tumours, for which the routine staining methods are sometimes unable to give a definite diagnosis.

### MATERIAL AND METHODS

The study has been based upon 101 cases of human benign and malignant soft tissue tumours. They were obtained from the Institute of Pathological Anatomy of the University of Pavia (Italy) and from the Hospital of Busto Arsizio (Italy), over a period of 10 years, since 1953. The majority of these came from surgical operations and only very few of them from autopsies. Only typical cases have been considered, the cases with doubtful diagnosis having been discarded. Our material was composed of tumours arising in the dermis in 95 per cent of the cases and has been divided, according to the classification proposed by Saphir (1959), into three main groups including vascular, mesodermal and nerve sheath tumours.

The collected cases are listed in Table I, together with the sex incidence and the age range for each histological type.

TABLE I.—*Sex Incidence and Age Range in Each Histological Type of Soft Tissue Tumours Collected*

| No. of cases | Group | Histological type | Sex incidence ♂ | ♀ | Age range (years) min. | max. | average |
|---|---|---|---|---|---|---|---|
| 30 | Vascular tumours | Haemangioma | 9 | 3 | 1 | 48 | 20·4 |
| | | Sclerosing haemangioma | 6 | 7 | 16 | 62 | 34·0 |
| | | Glomus tumour | 3 | 2 | 19 | 41 | 33·6 |
| 42 | Mesodermal tumours | Fibroma | 9 | 9 | 1 | 66 | 33·9 |
| | | Fibrosarcoma | 5 | 8 | 40 | 81 | 63·3 |
| | | Sarcoma | 6 | 5 | 1 | 73 | 43·8 |
| 18 | Nerve sheath tumours | Neurilemmoma | – | 5 | 36 | 60 | 48·6 |
| | | Neurofibroma | 5 | 8 | 2 | 68 | 44·2 |
| | | Lipoma | 1 | 5 | 69 | 26 | 48·0 |
| 11 | Miscellaneous | Rhabdomyosarcoma | 2 | 2 | 38 | 26 | 56·5 |
| | | Granular cell myoblastoma | 1 | 1 | 31 | 38 | 34·5 |

All the studied material has been fixed in 10 per cent buffered formalin, embedded in paraffin, histological diagnosis being determined by using the routine staining methods, i.e. haematoxylin and eosin, Mallory, Van Gieson and Sudan III on frozen sections. In order to investigate the relationship existing between mast cells and tumours additional sections were cut at 7 $\mu$ and stained with a 1 : 10·000 aqueous solution of toluidine blue buffered at pH 7·8 for 20 minutes, then, according to the technique proposed by Lascano (1958), decolorized with 200 c.c. of distilled water plus one drop of acetic acid, and next washed in 100 c.c. of distilled water plus one drop of ammonia water for one minute and mounted in glycerine.

The sections stained for the mast cell counts were microscopically examined, using a Hertell and Reuss Kassel microscope with a No. 6 ocular and a 45 objective. The same microscope and the same magnification were used throughout the study. Mast cells were counted in ten representative microscopic fields in each case, only considering the cells present in frankly neoplastic tissue. The mean number of mast cells for each histological type of tumour was determined, and the corresponding standard error (S.E.) calculated according to the following formula:

$$\sqrt{\frac{\Sigma (x - x)^2}{n(n - 1)}}$$

*Mice:* Random-bred Chester Beatty stock strain mice were used in Experiments II and IV. All mice were vaccinated on the tail with sheep lymph as a precaution against ectromelia. Injections were begun when the mice were 6 weeks old (25–35 g.). (Mice of this strain are exceptionally large.) Mice were housed in metal cages, 5 to a cage, fed Diet 41B and given water *ad libitum.*

*Preparation of ferritin.* The details of the preparation of ferritin are as follows :—

Two kilograms of rat's liver were collected and homogenised with water and heated to 80° C. to coagulate. The mass was then filtered through muslin and subsequently through a Whatman No. 1 filter paper. A clear brown solution was obtained.

To each 100 ml. of the above solution ammonium sulphate (A.R. grade) (30 g.) was added and the suspension allowed to stand at 4° C. in a refrigerator overnight. The resulting precipitate was collected by centrifugation. It was then dissolved in water and filtered, cadmium sulphate solution (4–5 g. per 100 ml.) added, and the whole kept at 4° C. in a refrigerator for 2 days. The resulting precipitate was collected by centrifugation and dried in a desiccator. The dried crude ferritin was re-crystallised by dissolving in ammonium sulphate solution (10 %) to which was added cadmium sulphate solution (4 %). After standing some time in the cold the ferritin was collected and rapidly washed with a saturated solution of potassium chloride.

The crystallisation of ferritin can be achieved by means of Zn, Cd, Ni or Co salts. Cadmium sulphate is the salt most commonly chosen for this. It seems necessary to have one of these elements present to form crystals. The cadmium may be considered to serve two functions ; the first, to co-ordinate the molecules of ferritin into a definite lattice pattern ; the second to decrease the solubility of ferritin, thus favouring crystallisation. The cadmium content of the re-crystallised ferritin was reduced by washing with a saturated potassium chloride solution, but some cadmium certainly remained in the final ferritin product used.

*Chemicals.* Crystalline cadmium sulphate with the formula $Cd\ SO_4 . 4H_2O$ was used, after a check had been made on its content of cadmium and water of crystallisation.

*Observation of animals.* Animals were examined regularly each week for the presence of tumours at the site of injection. They were killed when they became sick or developed rapidly growing injection-site tumours.

*Post-mortem examination.* All animals, whether killed because sick, or found dead, were subjected to careful post mortem examination. Organs showing pathological appearances were taken for histological examination.

### *Experiment I : Induction of Sarcomata at the Site of Subcutaneous Injection of Cadmium-precipitated Rat-Ferritin (in Rats)*

Twenty male rats, 3 weeks old, were injected subcutaneously in the right flank with ferritin prepared as above : the initial dose was 20 mg. but this caused severe ulceration at the injection site. A second dose of 20 mg. was given after an interval of 46 days and since this also caused a severe local reaction it was followed by eight doses of 2 mg. at weekly intervals, all given subcutaneously at the same site.

After 15 months one rat developed a palpable tumour at the site of injection for the lipomas ; no mast cells at all were counted in four cases of rhabdomyosarcoma and in two instances of granular cell myoblastoma.

The shape of the mast cells in all the groups studied was variable : oval, round, pear-shaped or elongated. The nuclei were oval and sometimes round, usually with a dense chromatin network. The nuclear cytoplasmic ratio was approximately 1 to $1\frac{1}{2}$. The metachromatic granules of mast cells were coarse and in some instances so dense that the nucleus was barely detectable. They frequently occurred in small groups. Mitotic figures were never observed.

### DISCUSSION

From our study an opportunity arises to compare the mast cell counts in the different soft tissue tumours collected. The general means for the three main groups are given ; it is clear that the maximum number of mast cells was found in the vascular tumours. A lower number occurred in the nerve sheath tumours and the lowest values were obtained for the mesodermal tumours. The facts mentioned above indicate that considering the number of mast cells present in the tumour tissue, a general differentiation should be possible for the various kinds of soft tissue tumours.

As far as the vascular tumours are concerned, the maximum mast cell values were found in the haemangiomas, i.e. in the tumours having the more pronounced vascular structure. If we compare the mast cell counts for the sclerosing haemangiomas and for the true fibromas, i.e. for two types of tumours histologically similar, we see that in the vascular type the mast cells were present in a noticeably greater number ; this difference in number was statistically highly significant. Actually, it is well established that mast cells tend to align themselves along arterioles or capillaries (Asboe-Hansen, 1950 ; Riley, 1953 ; Riley and West, 1953*a*, *b* and 1955 ; Kelsall and Crabb, 1959) ; on the other hand mast cells are assumed to increase in number in connection with the growth of connective tissue (Staemmler, 1921 ; Brack, 1925 ; Higuchi, 1930 ; Michels, 1938 ; Asboe-Hansen, 1950 ; Hjelmman, 1952 ; Telkä and Kunsisto, 1957 ; Lindholm, 1959). The present investigation, however, does not seem to confirm the latter assertion. Actually, our data seem to indicate that the mesodermal tumours are characterized by the presence of an inconspicuous number of mast cells, and this statement is confirmed by the analysis of the mast cell counts for the single types in which the mesodermal tumours have been histologically classified. Besides this, it is worthy of note that we have observed an inverse relationship between number of mast cells and histological malignancy. The fibromas have shown a higher number of mast cells than the fibrosarcomas and sarcomas, the latter tumours having a histological degree of malignancy which is progressively more evident.

Another question worth noting concerns the occurrence of mast cells in mesodermal and nerve sheath tumours. As previously shown, there is a clear cut difference between these two groups as far as the number of mast cells is concerned; thus providing a good differential diagnostic test for histologically similar tumours such as fibroma and neurofibroma. The fact that in the group of nerve sheath tumours in which the connective tissue stroma is more abundant, i.e. the neurofibromas, the number of mast cells is higher than in the neurilemmomas in which the connective stroma is less conspicuous, appears to be contradictory. At present we find this observation impossible to explain.

The additional soft tissue tumours we have collected such as lipomas, rhabdomyosarcomas, and granular cell myoblastomas are too limited in number to draw any definite conclusion. It is perhaps interesting to note that there is a complete absence of mast cells in all the cases of rhabdomyosarcoma and granular cell myoblastoma, and yet in the six instances of lipoma relatively high numbers were observed.

The role played by mast cells in relation to neoplasia has been discussed by many authors and is not dealt with in this research. The only conclusion we can definitely draw is that, on the basis of their mast cell content, it is possible to differentiate some types of soft tissue tumours, mainly the neurofibroma from the true fibroma, and the sclerosing haemangioma from the true fibroma. By this statement we do not want to diminish the importance of the routine histological methods, but we are just suggesting a reliable procedure which should be useful in finding out the exact histological diagnosis.

### SUMMARY

The purpose of the present investigation has been to study the occurrence of mast cells in various human soft tissue tumours mainly of vascular, mesodermal and nerve sheath origin. Haemangiomas, sclerosing haemangiomas, glomus tumours, true fibromas, fibrosarcomas, sarcomas, neurilemmomas and neurofibromas have been considered. Additionally a group of soft tissue tumours, lipomas, rhabdomyosarcomas and granular cell myoblastomas have also been studied. The mean mast cell count and the corresponding standard error have been calculated for each group. From the study of the above mentioned material the fact emerges that, according to their content of mast cells, it is possible to differentiate some of the investigated tumours, mainly true fibroma from sclerosing haemangioma and from neurofibroma.

I wish to express my gratitude to Professor C. Cavallero for his help in the preparation of this article.

### REFERENCES

Asboe-Hansen, G.—(1950) *Acta derm-venerol., Stockh.*, **30**, 338.
Bozzo, G. B.—(1953) *Quad. Clin. ostet. ginec.*, **8**, 657.
Brack, E.—(1925) *Folia haemat., Lpz.*, **31**, 202.
Bruni, C. and Olivi, M.—(1951) *Lav. Ist. Anat. Univ. Perugia*, **10**, 73.
Calonius, P. E. B. and Jäämeri, K. E. U.—(1961) *Ann. Chir. Gyn. Fenn.*, **50**, 9.
Cawley, E. P. and Hoch-Ligeti, C.—(1961) *Arch. Derm. Syph., N.Y.*, **83**, 92.
Corbetta, S.—(1951) *Arch. De Vecchi*, **17**, 31.
Cornil, L. and Michon, P.—(1924) *C.R. Soc. Biol., Paris*, **91**, 787.
Dunn, M. R. and Montgomery, P. O'. B.—(1957) *Lab. Invest.*, **6**, 542.
Higuchi, K.—(1930) *Folia haemat., Lpz.*, **41**, 401.
Hjelmman, G.—(1952) *Soc. Sci. Fenn. Comm. Biol.*, **13**, 10.
Janes, J. and McDonald, J. R.—(1948) *Arch. Path.*, **45**, 622.
Kelsall, M. A. and Crabb, E. D.—(1959) "Lymphocytes and Mast Cells". Baltimore (The Williams and Wilkins Co.), p. 85.
Lascano, E. F.—(1958) *Cancer* **11**, 1110.
Lindholm, S.—(1959) *Acta path. microbiol. scand.*, **46**, suppl. 132, 11.
Michels, N. A.—(1938) "Handbook of haematology" edited by H. Downey. New York (Paul B. Hoeber, Inc.), Vol. 1, p. 231.

PAVONE-MACALUSO, M.—(1960) *Arch. De Vecchi*, **33**, 193.
RILEY, J. F.—(1953) *J. Path. Bact.*, **65**, 471.
*Idem* AND WEST, G. B.—(1953*a*) *J. Physiol.*, **119**, 449.—(1953*b*) *Ibid.*, **120**, 528.—(1955) *J. Path. Bact.*, **69**, 269.
STAEMMLER, M.—(1921) *Frankfurt. Z. Path.*, **25**, 391.
STEINER, K.—(1961) *Arch. Derm. Syph., N.Y.*, **84**, 477.
TELKÄ, A., KUNSISTO, A —(1957) *Acta path. microbiol. scand.*, **36**, 304.
SAPHIR, O.—(1959) 'A text on Systemic Pathology'. New York and London (Grune and Stratton), Vol. II, pp. 1594–1618.